# Influence of EGFR-activating mutations on sensitivity to tyrosine kinase inhibitors in a KRAS mutant non-small cell lung cancer cell line

**DOI:** 10.1371/journal.pone.0229712

**Published:** 2020-03-04

**Authors:** Yoshinori Tsukumo, Mikihiko Naito, Takayoshi Suzuki

**Affiliations:** Division of Molecular Target and Gene Therapy Products, National Institute of Health Sciences, Kawasaki, Japan; H. Lee Moffitt Cancer Center & Research Institute, UNITED STATES

## Abstract

In non-small cell lung cancer (NSCLC), oncogenic driver mutations including those in *KRAS* and *EGFR* are typically mutually exclusive. However, recent reports indicate that multiple driver mutations are found in a certain percentage of cancers, and that the therapeutic responses of such cases with co-mutations of driver genes are largely unclear. Here, using CRISPR-Cas9-mediated genome editing, we generated isogenic cell lines harboring one or two copies of an EGFR-activating mutation from the human NSCLC cell line A549, which is known to harbor a homozygous *KRAS* gene mutation. In comparison with parent cells with *KRAS* mutation alone, cells with concomitant *EGFR* mutation exhibited higher sensitivity to EGFR-tyrosine kinase inhibitors (TKIs) but not to conventional anti-cancer drugs. In particular, cells with two copies of *EGFR* mutation were markedly more sensitive to EGFR-TKIs compared with parent cells. Thus, the presence of concomitant EGFR mutation can affect the TKI response of KRAS-mutated cells, implying that EGFR-TKI may represent an effective treatment option against NSCLC with EGFR/KRAS co-mutation.

## Introduction

Lung cancer is the leading cause of cancer-related death worldwide, and non-small cell lung cancer (NSCLC) is the most common type of lung cancer [1, 2]. Lung adenocarcinomas, which account for approximately 50% of NSCLC, are molecularly subclassified and subjected to different therapeutic strategies according to the presence of distinct alterations in genes such as *EGFR*, *ALK*, and *KRAS* [3]. Among such mutations, EGFR-activating mutation is that most frequently identified in Asian patients, accounting for 30%–50% of cases [4], while in Western patients, KRAS mutation is the most common, accounting for 25% of cases [3].

In NSCLC prior to treatment, the majority of oncogenic driver mutations are mutually exclusive with other mutations [5, 6]. However, recent studies have shown that additional driver mutations such as KRAS, ALK, and PI3K mutations co-exist with EGFR-mutations in a certain percentage of lung cancers [5, 7–14]. Of note, these driver mutations have been found not only in different cell populations in tumors, but also within the same cell population [15, 16]. However, little is known about the therapeutic responses of cancer cells harboring multiple driver mutations.

Cancer cells harboring EGFR-activating mutations have been shown to be highly sensitive to the EGFR tyrosine kinase inhibitor (TKI) gefitinib, which was first approved in Japan in 2002 [17, 18]. In addition to gefitinib, several EGFR-TKIs including afatinib and osimertinib have since been developed and approved for first-line treatment of advanced NSCLC harboring EGFR-activating mutations [17]. Unlike EGFR, however, effective drugs targeting KRAS have not been developed to date. Indeed, small molecules targeting the KRAS pathway, including farnesyl transferase inhibitors and downstream MEK inhibitors, have failed to show a beneficial effect, while KRAS mutant allelic imbalance is likely to influence the efficacy of MEK inhibitors [19]. Thus, there remains a significant unmet clinical need to address KRAS-mutated NSCLC, given the high frequency of cases having this mutation.

In the present study, we aimed to assess the response to EGFR-TKIs of cancer cells with EGFR/KRAS co-mutations. Using CRISPR/Cas9 genome editing, we successfully generated isogenic cell lines with one or two copies of L858R *EGFR* mutation, one of the most common types of EGFR mutation [4], from A549 cells, a well-established cell line harboring homozygous G12S *KRAS* mutation but wild-type *EGFR* [20–22]. Comparison of TKI sensitivity among these cell lines showed that EGFR/KRAS co-mutated cells were more sensitive than parent cells, indicating that EGFR-TKIs may represent a treatment option for cancers harboring EGFR/KRAS co-mutation.

## Materials and methods

### Cell culture

The human NSCLC cell line A549 was obtained from the National Cancer Institute (Frederick, MD) and isogenic cell lines were maintained in Dulbecco’s modified Eagle’s medium containing 10% fetal bovine serum and 100 μg/mL of kanamycin (Sigma-Aldrich, St. Louis, MO, US).

### Plasmids, single-stranded oligodeoxynucleotides (ssODNs), and reagents

Plasmid pSpCas9n-(BB)-2A-GFP (PX461) was purchased from Addgene (Watertown, MA, US) [23, 24]. To create convenient 5′ overhangs for single guide (sg)RNA cloning in our setting, we digested the plasmid with *Bbs*I restriction enzyme (NEB, Ipswich, MA, US) to insert phosphorylated and annealed oligo sets: set 1, (5′-CACCGTCTGTGATCTTGACATGCTG-3′ and 5′-AAACCAGCATGTCAAGATCACAGAC-3′) and set 2 (5′-CACCGGCTGGCCAAACTGCTGGGTG-3′ and 5′-AAACCACCCAGCAGTTTGGCCAGCC-3′). Long ssODNs were purchased from Sigma-Aldrich. Gefitinib was also from Sigma-Aldrich, and afatinib (BIBW2992) was from Cayman Chemical Company (Ann Arbor, MI, US). Cisplatin and taxol were purchased from Nippon Kayaku Co., Ltd. (Tokyo, Japan).

### Transfection

Plasmids were transfected into cells by electroporation using the Neon Transfection System according to the manufacturer’s instructions (Thermo Fisher Scientific, Waltham, MA, US). In brief, 5 μg of pSpCas9n-(BB)-2A-GFP plasmids (2.5 μg of each plasmid with a different sgRNA sequence; set 1 and 2) were transfected into 5 × 10^5^ cells at 1550 V in a triple 10-ms pulse. For knock-in, ssODNs were dissolved in filtered water and transfected at 0.01 nmol per reaction together with 5 μg of the plasmids. After transfection, cells were incubated in the presence of 1 μM SCR-7, a non-homologous end joining inhibitor (Xcessbio, San Diego, CA, US), for 4 days.

### Positive clone selection using L858R mutation-specific primers

To isolate L858R knock-in clones, transfected cells were seeded at 1 cell/well into 96-well plates. After cells reached 50%–90% confluency, cell suspensions collected from each well were pooled into one tube (16 wells/tube), giving a total of 90 cell mixtures from 15 96-well plates. PCR analysis amplified a band corresponding to the expected size (177 bp) in 22/90 mixtures. For the second screening, each candidate clone was scaled up to growth in 24-well plates. A total of 21 candidate clones were obtained and further screened by immunoblotting using an L858R EGFR antibody (Cell Signaling Technology, Danvers, MA, US) and by sequencing around the target region.

### DNA extraction

Genomic DNA was extracted from cells by washing them with 200 μl phosphate-buffered saline and incubating them in 50 μl of 25 mM NaOH/0.2 mM EDTA solution for 10 min at 98°C. After cooling to room temperature, the lysates were mixed with 50 μl of 40 mM Tris-HCl. The lysate was then centrifuged at 4000 × g for 3 min prior to PCR genotyping using 2 μl of supernatant as a template. For DNA sequencing, genomic DNA was extracted using the DNeasy Blood & Tissue Kit (Qiagen, Germantown, MD, US).

### PCR genotyping and clone selection

To detect knock-in mutations, HiDi DNA Polymerase (myPOLS, Konstanz, Germany, US) was used for PCR. For positive clone selection, L858R mutation-specific primers were used: forward, 5′-ATTTTGGGCGGGCTAAGT-3′ and reverse, 5′-CCAGAATGTCTGGAGAGCAT-3′. DNA amplification was performed under the following conditions: 30 cycles of 95 °C for 30 sec, 58 °C for 15 sec, and 72 °C for 20 sec. Positive clones were incubated for 2–3 weeks, and extracted DNA was sequenced following subcloning into a sequence vector pcDNA3.1 (Thermo Fisher Scientific). For sequencing, target regions were amplified using the following primers: forward, 5′-CCTCACAGCAGGGTCTTCTC-3′ and reverse, 5′-ATCCTGCAGGGAGAGACTGA-3′. PCR products were directly sequenced with the forward primer.

### Copy number analysis

Genomic DNA was extracted using the DNeasy Blood & Tissue Kit (Qiagen). Quantitative real-time PCR was performed with an ABI Prism 7300 sequence detection system (Applied Biosystems, Foster City, CA, US) using SYBR GreenER (Invitrogen) with EGFR-specific primers: EGFR_ex27 forward, 5′-GCCTTGACTGAGGACAGCAT-3′ and EGFR_ex27 reverse, 5′-TACGCCCTTCACTGTGTCTG-3′. The relative copy number to the control in TIG-3 (diploid) and A549 (triploid) cells was calculated using the comparative Ct method.

### mRNA expression analysis

Total RNA was prepared from cells using an RNeasy mini kit (Qiagen). First-strand cDNA was synthesized from 1 μg total RNA with an oligo-dT primer using the SuperScript III First-Strand Synthesis System (Invitrogen). The following PCR primers were used: ACTB forward, 5′-AGAGCTACGAGCTGCCTGAC-3′ and reverse, 5′-AGCACTGTGTTGGCGTACAG-3′; total EGFR forward, 5′-CTGGATCCCAGAAGGTGAGA-3′ and reverse, 5′-GTCTTTGTGTTCCCGGACAT-3′; EGFR wild-type forward, 5′-ATTTTGGGCTGGCCAAAC-3′ and reverse, 5′-GATTCCGTCATATGGCTTGG-3′; and EGFR mutant forward, 5′-ATTTTGGGCGGGCTAAGT-3′ and reverse, 5′-GATTCCGTCATATGGCTTGG-3′. Band intensity was quantified using Image J software [25].

### Immunoblotting

Cells were lysed with sodium dodecyl sulfate (SDS) lysis buffer (62.5 mM Tris-HCl, pH 7.2, 10% glycerol, 1% SDS) and immediately boiled for 10 min to obtain clear lysates. The protein concentration was measured by the Bradford method (BIO-RAD, Hercules, CA, US), and lysates containing an equal amount of proteins were separated by SDS–polyacrylamide gel electrophoresis and transferred to polyvinylidene fluoride membranes (Millipore, Darmstadt, Germany) for western blot analysis using appropriate antibodies. Immunoreactive proteins were visualized using the Immobilon Western Chemiluminescent HRP substrate (Millipore) or Clarity Western ECL substrate (BIO-RAD), and light emission was quantified with a LAS-3000 lumino-image analyzer (Fuji, Tokyo, Japan). The antibodies used in this study were: anti-EGFR, phospho-EGFR (Y1068), L858R EGFR, AKT, and phospho-AKT(S473) (Cell Signaling Technology, Danvers, MA, US) and anti-β-actin (Sigma-Aldrich). Band intensity was quantified using Image J software.

### Cell viability assay

Cell viability was determined using water-soluble tetrazolium WST-8 (4-[3-(2-methoxy-4-nitrophenyl)-2-(4-nitrophenyl)-2H-5-tetrazolio]-1,3-benzene disulfonate) for the spectrophotometric assay according to the manufacturer’s instructions (Dojindo, Tokyo, Japan). Cells were plated at 1 × 10^3^ cells/well in 96-well plates and treated with gefitinib (10–1000 nM) and afatinib (0.3–30 nM) for 96 h, or cisplatin (0.3–100 μM) and taxol (0.03–10 nM) for 72 h. Following drug treatment, cells were incubated with WST-8 reagent for 30 min at 37°C in a humidified atmosphere of 5% CO_2_. The absorbance at 450 nm of the medium was measured using an EnVision Multilabel Plate Reader (PerkinElmer, Waltham, MA, US).

### Colony formation assay

Cells were plated in 6-well plates at 200 cells per well. After 1 day, they were incubated in the presence or absence of the indicated concentration of TKIs gefitinib and afatinib. Fresh media containing each TKI was added every 3 days. After 10 days, cells were fixed in methanol and stained with 0.5% crystal violet. The number of foci >1 mm was automatically determined using Image J software.

### Statistical analysis

Error bars for all data represent standard deviation (s.d.) from the mean. P-values were calculated using Bonferroni correction for multiple comparisons using online statistical analysis tool (https://astatsa.com), as shown in each figure legend.

## Results

### Design and preparation of tools for CRISPR-Cas9 genome editing

**Fig 1:**
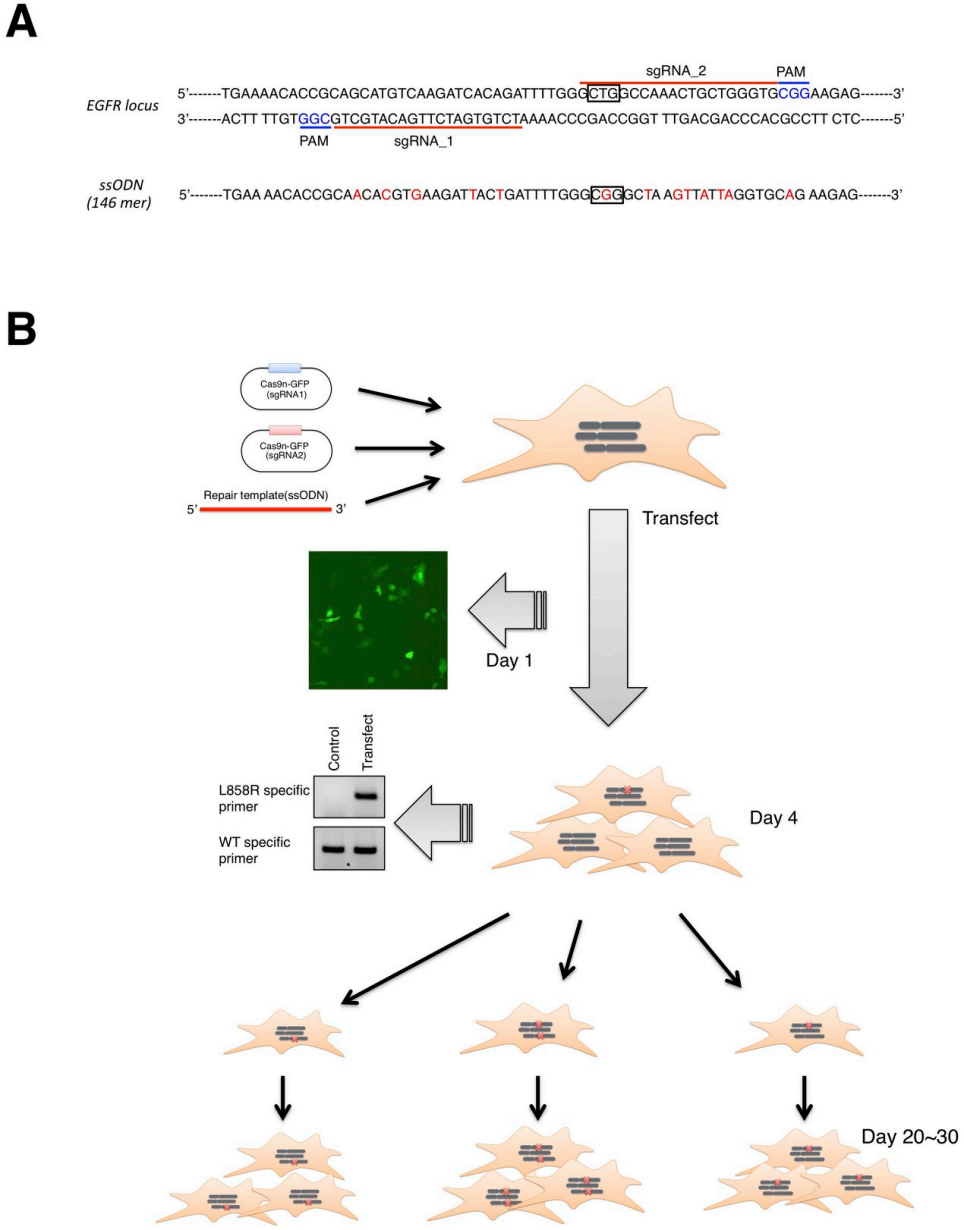
Overview of CRISPR/Cas9 genome editing. **A.** Sequence of human *EGFR* exon 21 for CRISPR/Cas9 genome editing. sgRNA target sites and protospacer adjacent motifs are indicated by red bars and blue characters, respectively. The ssODN sequence as a repair template is also shown. Box represents Leu or Arg residue at amino acid position 858. **B.** Timeline of genome editing. Cas9n (sgRNA) plasmids and ssODN for homology directed recombination (HDR) are transfected into A549 cells. Successful transfection and HDR were validated by the presence of GFP-positive cells at day 1 and a mutation-specific PCR product at day 4, respectively. Transfected cells were clonally expanded to isolate L858R-knockin clones.

Using the CRISPR Design Tool (http://crispr.mit.edu), we designed sgRNAs targeting leucine 858 in *EGFR* exon 21 (Fig 1A). For genome editing, we chose the CRISPR-Cas9D10A nickase (Cas9n) system, which minimizes off-target activity [23, 24, 26]. The human NSCLC cell line A549, which contains three copies of wild-type *EGFR* [27], was co-transfected with two *EGFR*-targeting Cas9n vectors and a ssODN (146 nucleotides, sense strand) donor containing a CTG→CGG mutation (boxed in Fig 1A) flanked by sequences homologous to the *EGFR* target site. Importantly, we synonymously mutated one nucleotide in the protospacer adjacent motif and five or six nucleotides in the ‘seed’ region (3′ region) of the sense or anti-sense sgRNA sequence to prevent repetitive attack by Cas9n after successful integration of the exogenous sequence (Fig 1A) [26]. Four days after transfection, we confirmed the presence of the L858R mutation within *EGFR* exon 21 in transfected cells by PCR using L858R mutation-specific primers (Fig 1B). Three clones were shown to harbor the L858R mutation (clones 82–12, 47–3, and 73–4). There was no significant difference in cell growth between mutant clones and parental cells under normal culture conditions (S1 Fig).

### Establishment of cell lines harboring the L858R *EGFR* mutation

**Fig 2:**
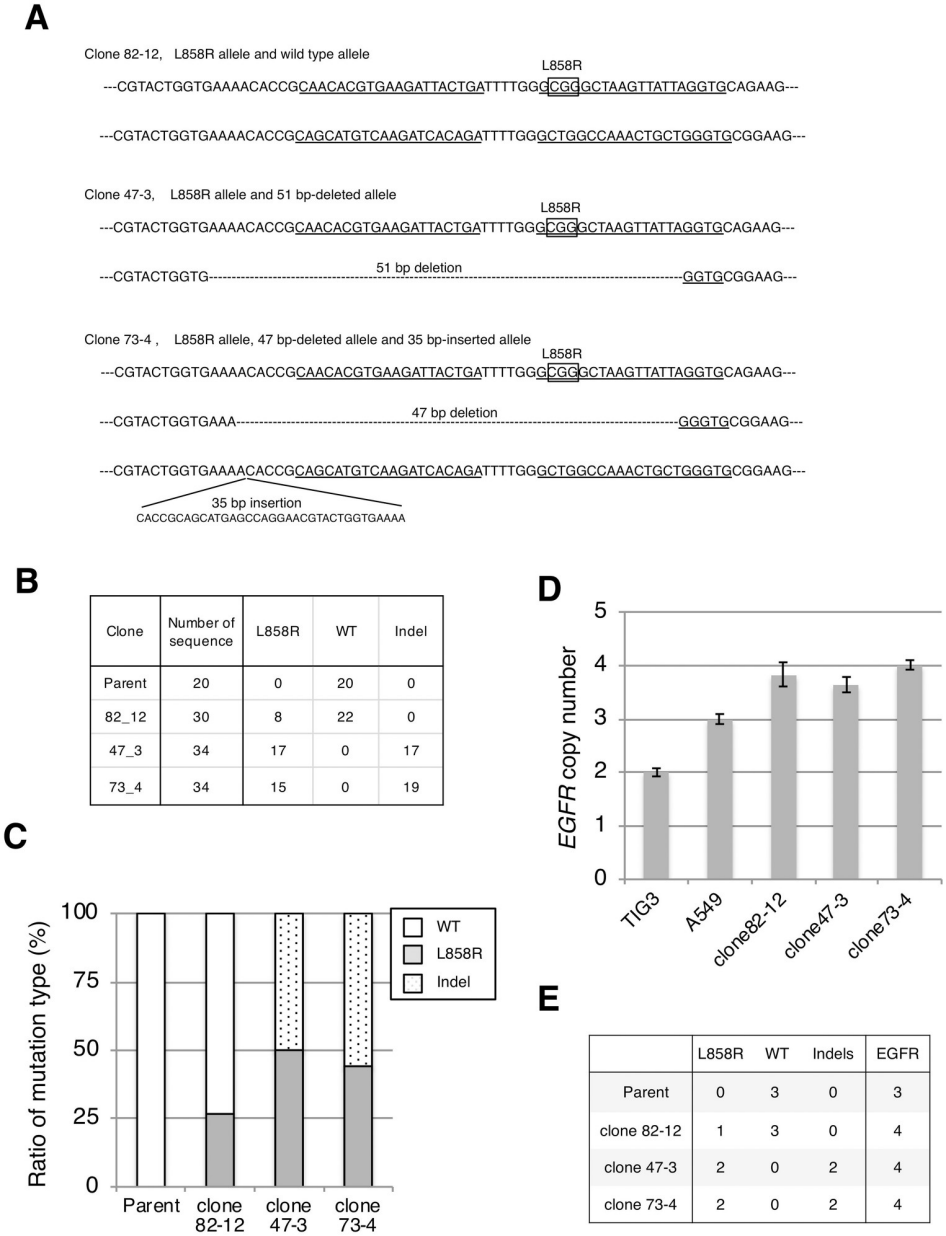
Establishment of cell lines harboring the L858R *EGFR* mutation. **A.** Sequence of *EGFR* target region in each clone. Sanger sequencing was performed after subcloning the PCR-amplified target region into a plasmid vector. Box: L858R mutation (CTG→CCG), underline: sgRNA target regions. **B.** Summary of the number and type of each sequence. **C.** The mutation ratio of L858R or deletion (or insertion) was calculated based on the results shown in B. **D.** The *EGFR* copy number was determined by quantitative PCR using the diploid cell TIG3 as a control. **E.** The L858R *EGFR* copy number was calculated from the mutation ratio of L858R (**C**) and *EGFR* copy number (**D**).

To confirm successful integration of the L858R mutation, the targeted genomic region was PCR-amplified and subcloned into a plasmid vector, and 20–30 bacterial colonies were sequenced for each clone. Sequence analysis showed that clone 82–12 contained both the L858R allele (boxed CGG) and the wild-type allele (Fig 2A). Clones 47–3 and 73–4 carried L858R alleles but no wild-type allele, instead carrying *EGFR* alleles with a 51 bp deletion (clone 47–3) or 47 bp deletion and 35 bp insertion (clone 73–4) (Fig 2A). We calculated the L858R mutation ratio as 26.7% (8/30) for clone 82–12 and approximately 50% (17/34 or 15/34) for clones 47–3 and 73–4 (Fig 2B and 2C).

Next, we investigated the *EGFR* copy number in each clone by quantitative PCR. Comparison with the normal human diploid cell line TIG-3 indicated that each clone carried nearly four copies of *EGFR*, while parental A549 cells had three copies as reported previously (Fig 2D) [27]. From the results of the L858R mutation ratio (Fig 2C) and *EGFR* copy number (Fig 2D), clones 82–12, 47–3, and 73–4 were inferred to have one, two, and two copies of the L858R *EGFR* mutation, respectively (Fig 2E).

### Expression of the L858R *EGFR* mutant in established clones

**Fig 3:**
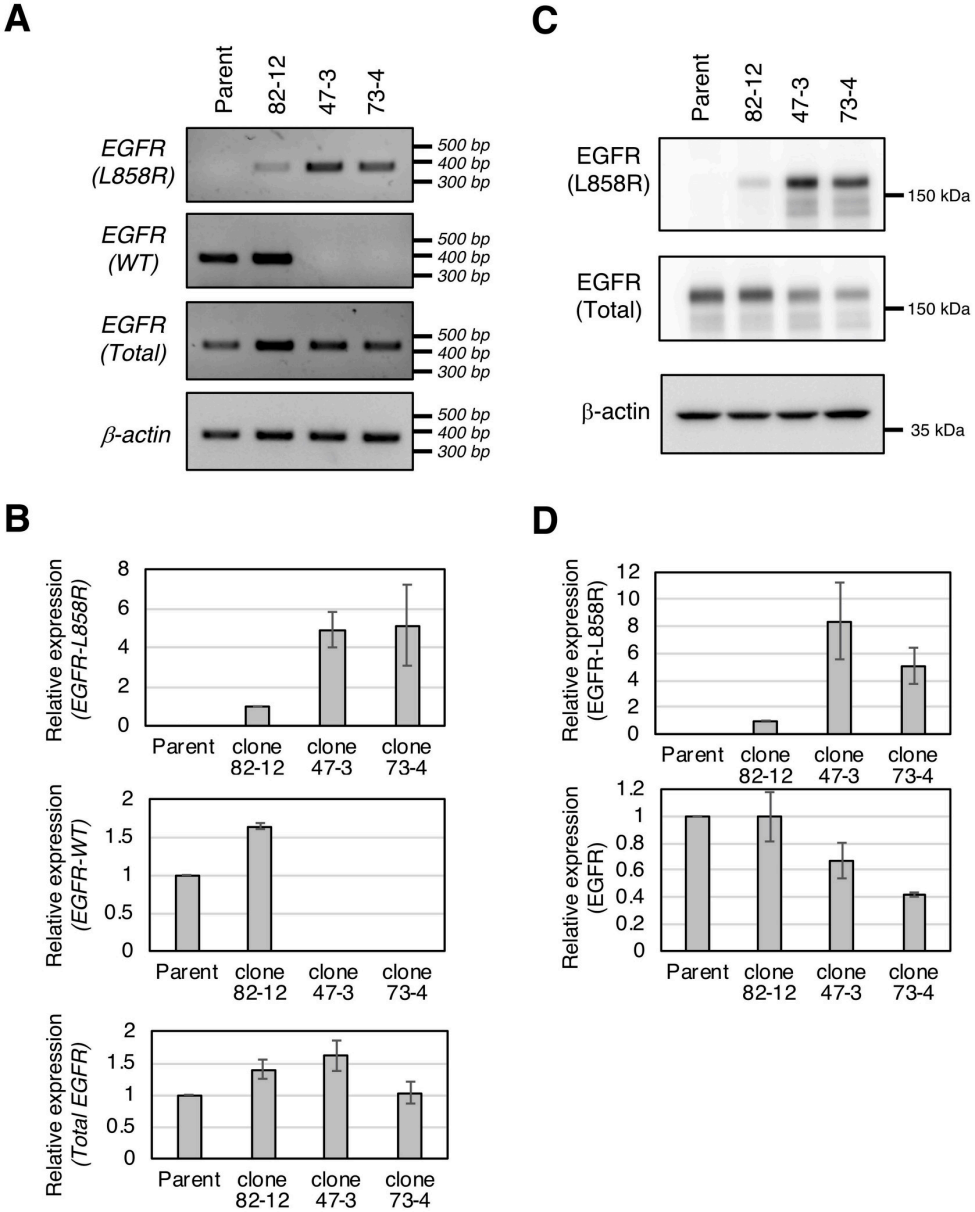
Expression of the L858R *EGFR* mutant in established clones. **A.** L858R *EGFR*, wild-type *EGFR*, and total *EGFR* mRNA expression was analyzed by PCR using specific primer sets. **B.** mRNA expression was normalized to β-actin. Densitometric analysis of each band was performed by Image J software. For L858R *EGFR* mRNA, the expression in clone 82–12 was used as a control; for wild-type or total *EGFR* mRNA, parental expression was used as a control. **C.** L858R EGFR or total EGFR protein expression was analyzed by immunoblotting with an L858R-specific or anti-EGFR antibody. **D.** L858R EGFR or total EGFR protein was quantified as shown in **B**.

We examined the expression of the L858R *EGFR* mutant in each clone at both the mRNA and protein level. First, we measured wild-type *EGFR*, L858R *EGFR*, and total *EGFR* mRNA expression using allele-specific primers (Fig 3A). Consistent with the genomic analysis (Fig 2), wild-type *EGFR* mRNA was expressed in parental cells and clone 82–12 but not in clones 47–3 or 73–4, while L858R *EGFR* mRNA was detected in all three genome-edited clones, with higher levels in clones 47–3 and 73–4 than in clone 82–12 (Fig 3A and 3B). The expression of total *EGFR* mRNA in clone 73–4 was lower than in clone 47–3 (Fig 3A and 3B). Of note, *EGFR* mRNAs containing a frame-shift (35 nt insertion or 47 nt deletion) in clone 73–4 were barely expressed, while the mRNA containing in-frame 51 nt deletion in clone 47–3 was clearly expressed (S2 Fig). Therefore, the lower total *EGFR* mRNA expression in clone 73–4 may be attributable to degradation of mutant mRNAs by nonsense-mediated decay (NMD). Fig 3c and 3d show that the protein level of L858R EGFR in each clone correlated well with L858R *EGFR* mRNA expression. However, the expression of total EGFR protein in clones 47–3 and 73–4 was lower than in parental cells, suggesting the possibility that the mutant EGFR protein encoded by the indel mutants had been eliminated by a protein quality control system such as the endoplasmic reticulum-associated degradation (ERAD) pathway.

### The influence of L858R *EGFR* mutant copy number on EGFR-TKI sensitization

**Fig 4:**
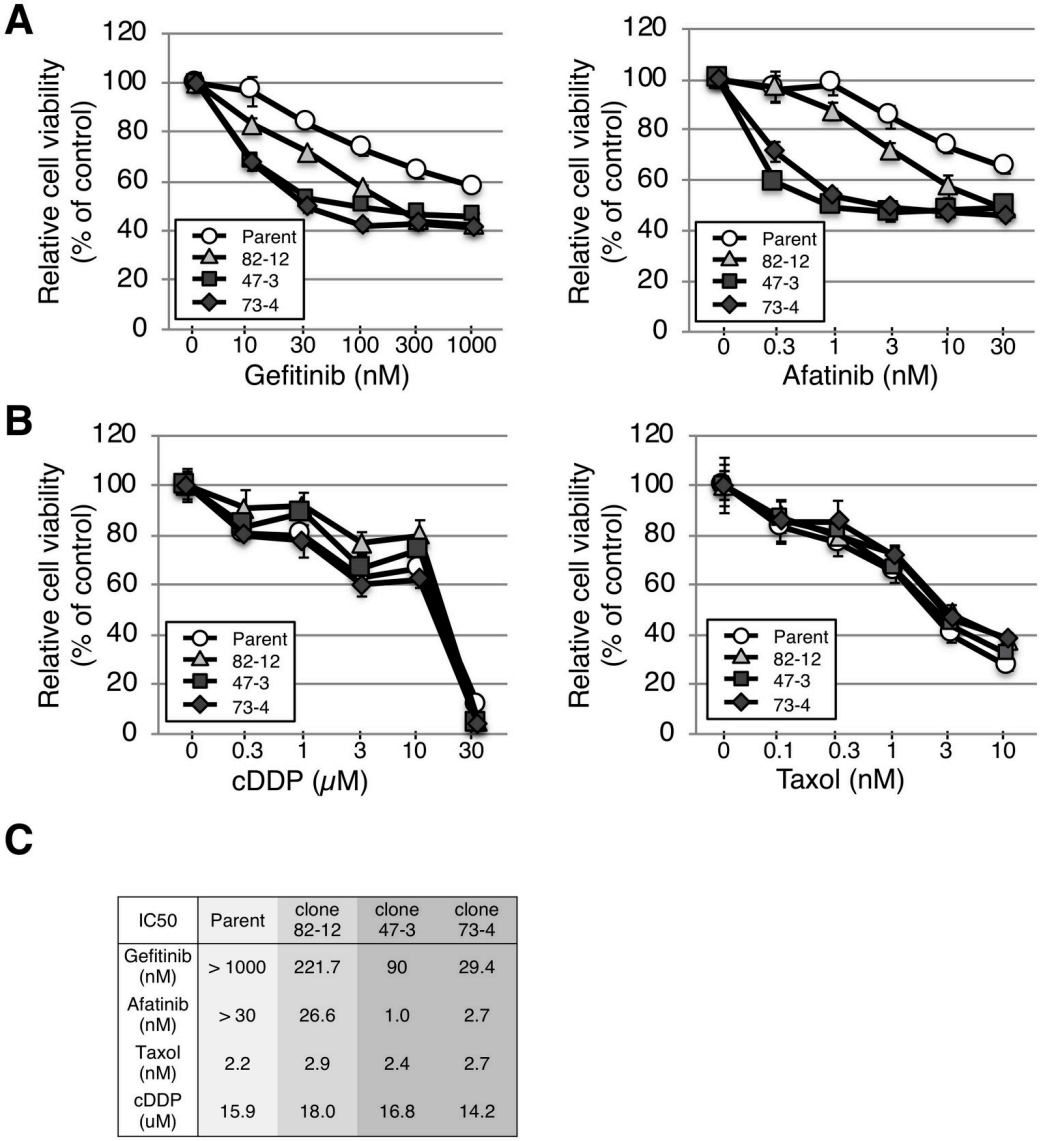
L858R *EGFR* mutation renders A549 cells sensitive to EGFR-TKIs. **A, B.** Cells were plated at 1 × 10^3^ cells/well in 96-well plates and treated with gefitinib (10–1000 nM) and afatinib (0.3–30 nM) for 96 h, or cisplatin (0.3–100 μM) and taxol (0.03–10 nM) for 72 h. Cell viability was evaluated using a WST assay. Each experiment was repeated three times. **C.** The IC50 of each drug (gefitinib, afatinib, taxol, and cisplatin) is shown.

We next examined the response of the cells to EGFR-TKIs (gefitinib and afatinib) and conventional anti-cancer drugs (cisplatin and taxol), which are standard treatment options in NSCLC [28, 29]. All three clones were more sensitive to EGFR-TKIs than parental cells (Fig 4A), indicating that one or two copies of the L858R mutation was sufficient to render cells sensitive to EGFR TKIs, as observed in cells with amplified *EGFR*-mutant genes [18, 30, 31]. Intriguingly, clones 47–3 and 73–4 with two copies of the L858R mutation showed approximately 2.5- and 7-fold higher sensitivities to gefitinib and 25- and 10-fold higher sensitivities to afatinib than clone 82–12 with only one copy of L858R (Fig 4C). The sensitivity of clones 47–3 and 73–4 was comparable to that of HCC827, which harbors the amplified EGFR-activating mutant allele (del 19), but not to TKI-resistant cell line H1975, suggesting that EGFR-activating mutation contributes to TKI sensitivity even against a background of KRAS mutation (S3A and S3B Fig). However, each clone and parental cell line exhibited similar sensitivities to the conventional anti-cancer drugs cisplatin and taxol (Fig 4B and 4C).

**Fig 5:**
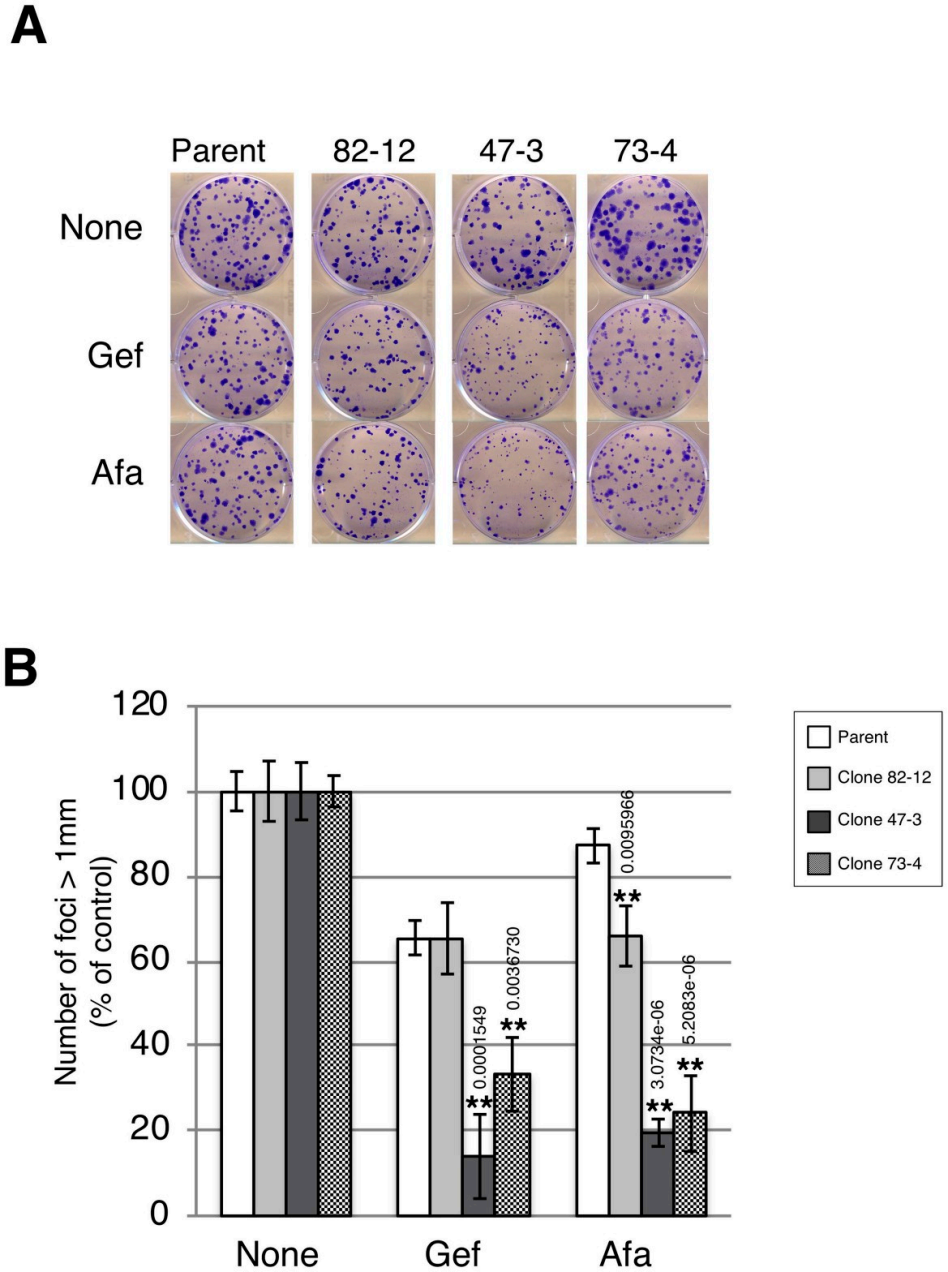
Two copies of L858R *EGFR* hypersensitize A549 cells to EGFR-TKIs. **A.** Clonogenic survival assay. Each cell line was plated at 200 cells/well in 6-well plates and incubated in the presence of gefitinib (10 nM) or afatinib (0.3 nM) for 10 days. Colonies were fixed by methanol and stained with crystal violet. **B.** The number of foci >1 mm was automatically counted using Image J software. Results are the mean cell number relative to control (set to 100%) ± SD (n = 3). Statistical analysis: Bonfferoni correction was used for multiple comparisons. *P<0.05, **P<0.01.

We further investigated the growth of each clone by the clonogenic survival assay in the presence of low-dose gefitinib (10 nM) and afatinib (0.3 nM) treatment. Afatinib treatment reduced the colony formation of each clone by 15% (parental), 35% (clone 82–12), 80% (clone 47–3), and 80% (clone 73–4) (Fig 5A and 5B). Similarly, strong growth inhibition was observed in gefitinib-treated clones with two copies of the L858R mutation (Fig 5A and 5B). Thus, clones with two copies of the L858R mutation were hypersensitive to TKIs compared with the clone with only one copy of the L858R mutation.

**Fig 6:**
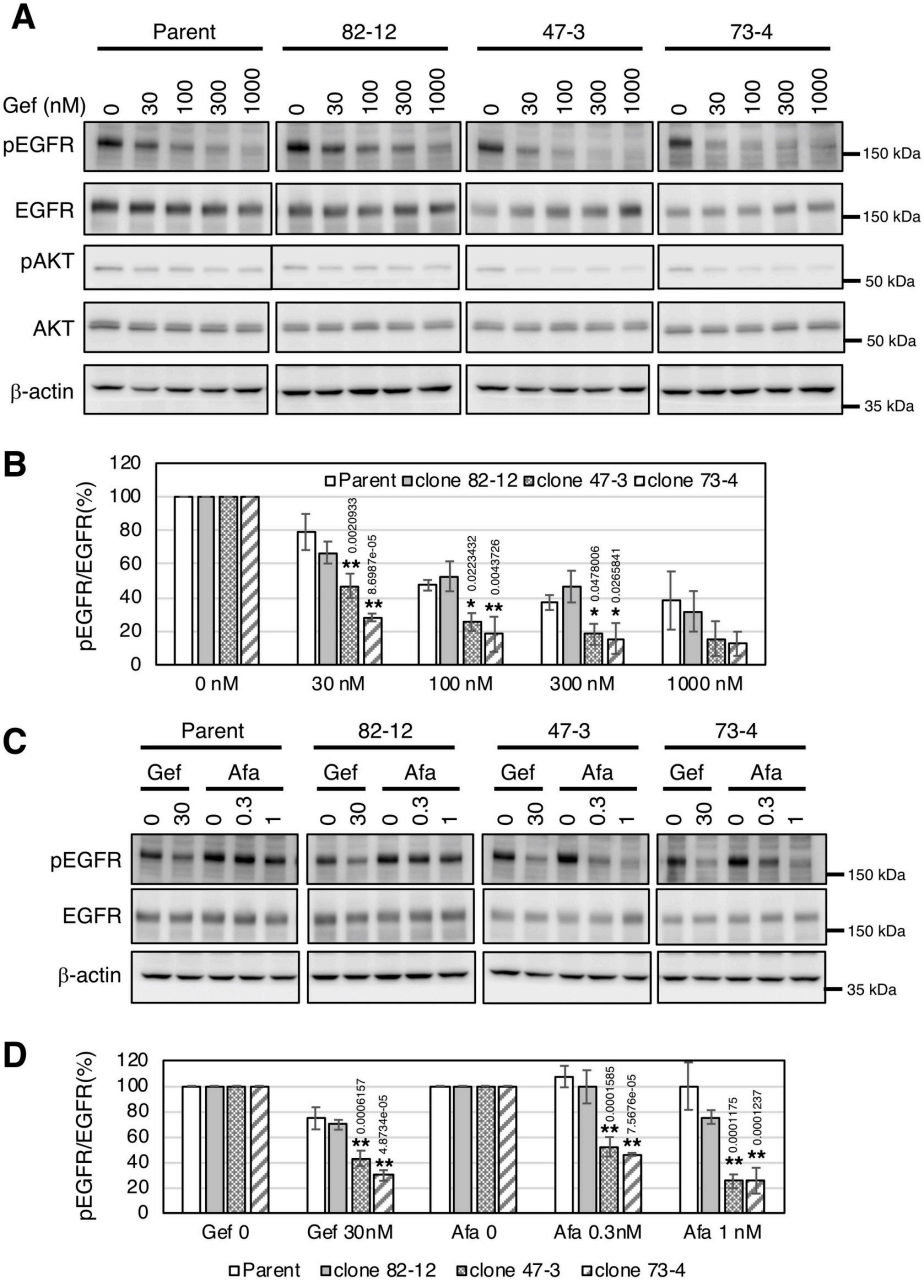
EGFR phosphorylation is markedly reduced by TKI treatment in cells with two copies of L858R *EGFR*. **A.** Cells were treated with the indicated concentration of gefitinib for 4 h. Protein expression or phosphorylation levels were determined by immunoblotting using anti-phospho-EGFR (Y1068), anti-EGFR, anti-phospho-AKT (Ser473), anti-AKT, and β-actin antibodies. **B.** Densitometric analysis of phospho-EGFR and EGFR protein was performed by Image J software. The value of pEGFR/EGFR in the non-treated sample lane was defined as 100%. *P<0.05, **P<0.01. **C.** Cells were treated with the indicated concentration of afatinib or gefitinib for 4 h. Protein expression or phosphorylation levels were determined by immunoblotting using anti-phospho-EGFR (Y1068), anti-EGFR, and β-actin antibodies. **D.** Densitometric analysis of phospho-EGFR and EGFR protein was performed by Image J software. The value of pEGFR/EGFR in the non-treated sample lane was defined as 100%. Statistical analysis: Bonfferoni correction was performed for multiple comparisons. **P<0.01.

Finally, we investigated the EGFR phosphorylation status in each clone in the presence of gefitinib or afatinib. Gefitinib treatment decreased EGFR phosphorylation in a dose-dependent manner in each cell (Fig 6A and 6B). EGFR phosphorylation was decreased with a lower dose of gefitinib treatment in clones 47–3 and 73–4 compared with parental cells (Fig 6A and 6B). Similarly, downstream AKT phosphorylation was clearly decreased in clones 47–3 and 73–4. However, no significant difference was found in EGFR phosphorylation between parental cells and clone 82–12 (Fig 6A and 6B). EGFR phosphorylation in clones 47–3 and 73–4, but not in parental cells or clone 82–12, was significantly reduced with afatinib treatment (p<0.01) (Fig 6C and 6D). Thus, EGFR phosphorylation in clones 47–3 and 73–4 harboring two copies of the L858R mutation was markedly inhibited with lower doses of TKIs, which is consistent with the results of growth inhibition shown in Figs 4 and 5.

## Discussion

In NSCLC, oncogenic driver mutations are typically mutually exclusive, although cases of mutations in multiple driver genes are increasingly reported [5, 7–14]. EGFR/KRAS co-mutation is likely to represent a certain proportion of cases of multiple mutations in NSCLC [12]. In the clinical setting, the limited prevalence of EGFR/KRAS co-mutated NSCLC means that its response to EGFR-TKI remains unclear. So far, several studies have reported the therapeutic effects of EGFR-TKI in patients with the co-mutations [9, 12, 32, 33]. Zhang et al. reported that EGFR/KRAS co-mutation was detected in 38 out of 2106 patients (1.8%), and that a larger proportion of patients with the co-mutation obtained a better objective response rate (38.9% vs. 9.5%) and longer progression-free survival (8.0 vs. 1.5 months) following EGFR-TKIs therapy compared with patients having a KRAS mutation alone. However, these differences were not observed in patients treated with platinum-based chemotherapy [33]. Likewise, several other studies have described cases in which EGFR/KRAS co-mutation responded to EGFR-TKI therapy [9, 12, 32]. Taken together, these reports indicate that EGFR-TKI therapy may represent an effective treatment strategy against NSCLC with EGFR/KRAS co-mutation.

Clinically, it is difficult to determine whether multiple driver mutations detected in tumors are derived from different cells or from the same cell population. Although both cases have been assumed and reported [15–17], the available information is limited, particularly for the latter. In this regard, we have successfully generated the first example of a cancer cell model with double driver mutations. Using the CRISPR/Cas9 system, we generated isogenic cell lines with EGFR/KRAS co-mutations from the KRAS-mutated A549 cell line (Figs 1 and 2), and demonstrated that these co-mutated cell lines were more susceptible to EGFR-TKIs, but not to conventional anti-cancer drugs, than parental cells (Fig 4). This was rather surprising, because KRAS is a signaling mediator downstream of EGFR and KRAS mutation was reported to be involved in TKI-resistance [34–37]. The dominant role of EGFR mutation over the KRAS mutation on the sensitivity to TKIs was supported by the result that HCC827 harboring EGFR mutation (ex19 dels) alone showed similar IC50 value to EGFR/KRAS co-mutated cells generated from A549 (S3 Fig). It should be noted that EGFR del 19 mutant is prone to be sensitive to TKIs compared to L858R mutant [38, 39]. To confirm these results, it is important to compare the sensitivity to EGFR-TKIs of the cell lines harboring EGFR mutation alone, KRAS mutation alone, and EGFR/KRAS co-mutations under the same genetic background.

Interestingly, clones with two copies of EGFR mutation (clones 47–3 and 73–4) harbored three and four copies of KRAS mutation, respectively (S4 Fig) and clone 73–4 with four copy KRAS mutation was prone to be resistant to gefitinib but not afatinib in a clonogenic survival assay (Fig 5B). In line with this, it was recently reported that NSCLCs with KRAS mutation transiently respond to a first-generation TKI, whereas they escape prolonged suppression through the activation of other ERBB family members while the second-generation TKI afatinib persistently suppresses tumor growth via broad inhibitory effects on the ERBB family [40, 41]. These observations suggest that although EGFR mutation plays a primary role on the sensitivity to EGFR-TKIs, co-mutation of KRAS also affects the sensitivity to TKIs.

In summary, our findings demonstrated that the coexistence of *EGFR* mutation in KRAS-mutated cells enhances TKI sensitivity, especially in the presence of a higher *EGFR* mutation copy number. Given that no effective targeted therapy against KRAS-mutated NSCLC is currently available, the identification and quantification of druggable mutations occurring concomitantly in tumors may provide an opportunity for the development of alternative treatment options for affected patients.

## Supporting information

S1 Fig Relative cell proliferation under normal growth condition. Cells were seeded at 100000 cells/well in 6-well plate. The numbers of viable cells were stained by trypan blue and counted at day 0 (next day), 1, 2, and 3.
(PDF)

S2 Fig Expression of indel form of EGFR mRNA in clone 47–3. Expressions of *EGFR* mRNAs containing intact and indel forms were analyzed by PCR using specific primer sets, which were designed around indel regions. Arrow: predicted PCR product, arrow head: 51 nt-deleted product.
(PDF)

S3 Fig TKI sensitivity of HCC827 and H1975 cells. **A.** Cells were plated at 1 ´ 10_3_ cells/well in 96-well plates and treated with gefitinib (10 to 1000 nM) and afatinib (0.3 to 30 nM) for 72 h. Cell viability was measured by the WST assay. **B.** The IC50 of gefitinib and afatinib is shown. **C.** HCC827 or H1975 cells were treated with the indicated concentration of gefitinib for 4 h. Protein expression was determined by immunoblotting using anti-phospho-EGFR (Y1068), anti-EGFR, and β- actin antibodies.
(PDF)

S4 Fig Copy number of mutant KRAS in clones. **A.** Homozygous KRAS G12S mutation in each clone was validated by Sanger sequence. **B.** The *KRAS* copy number was determined by quantitative PCR using the diploid cell TIG3 as a control.
(PDF)

S1 Methods (PDF)

## References

[1] Siegel RL, Miller KD, Jemal A. Cancer Statistics, 2017. CA: a cancer journal for clinicians. 2017;67(1):7–30. 10.3322/caac.21387 . 28055103

[2] Torre LA, Bray F, Siegel RL, Ferlay J, Lortet-Tieulent J, Jemal A. Global cancer statistics, 2012. CA: a cancer journal for clinicians. 2015;65(2):87–108. 10.3322/caac.21262 . 25651787

[3] Dearden S, Stevens J, Wu YL, Blowers D. Mutation incidence and coincidence in non small-cell lung cancer: meta-analyses by ethnicity and histology (mutMap). Annals of oncology: official journal of the European Society for Medical Oncology. 2013;24(9):2371–6. Epub 2013/06/01. 10.1093/annonc/mdt205 23723294 PMC3755331

[4] Mitsudomi T, Yatabe Y. Mutations of the epidermal growth factor receptor gene and related genes as determinants of epidermal growth factor receptor tyrosine kinase inhibitors sensitivity in lung cancer. Cancer science. 2007;98(12):1817–24. 10.1111/j.1349-7006.2007.00607.x . 17888036 PMC11159145

[5] Cancer Genome Atlas Research N. Comprehensive molecular profiling of lung adenocarcinoma. Nature. 2014;511(7511):543–50. Epub 2014/08/01. 10.1038/nature13385 25079552 PMC4231481

[6] Jamal-Hanjani M, Wilson GA, McGranahan N, Birkbak NJ, Watkins TBK, Veeriah S, et al Tracking the Evolution of Non-Small-Cell Lung Cancer. The New England journal of medicine. 2017;376(22):2109–21. 10.1056/NEJMoa1616288 . 28445112

[7] Baldi L, Mengoli MC, Bisagni A, Banzi MC, Boni C, Rossi G. Concomitant EGFR mutation and ALK rearrangement in lung adenocarcinoma is more frequent than expected: report of a case and review of the literature with demonstration of genes alteration into the same tumor cells. Lung Cancer. 2014;86(2):291–5. Epub 2014/10/15. 10.1016/j.lungcan.2014.09.011 . 25312989

[8] Blakely CM, Watkins TBK, Wu W, Gini B, Chabon JJ, McCoach CE, et al Evolution and clinical impact of co-occurring genetic alterations in advanced-stage EGFR-mutant lung cancers. Nat Genet. 2017;49(12):1693–704. Epub 2017/11/07. 10.1038/ng.3990 29106415 PMC5709185

[9] Guibert N, Barlesi F, Descourt R, Lena H, Besse B, Beau-Faller M, et al Characteristics and Outcomes of Patients with Lung Cancer Harboring Multiple Molecular Alterations: Results from the IFCT Study Biomarkers France. Journal of thoracic oncology: official publication of the International Association for the Study of Lung Cancer. 2017;12(6):963–73. Epub 2017/02/13. 10.1016/j.jtho.2017.02.001 . 28189832

[10] Hu W, Liu Y, Chen J. Concurrent gene alterations with EGFR mutation and treatment efficacy of EGFR-TKIs in Chinese patients with non-small cell lung cancer. Oncotarget. 2017;8(15):25046–54. Epub 2017/02/18. 10.18632/oncotarget.15337 28212572 PMC5421908

[11] Jordan EJ, Kim HR, Arcila ME, Barron D, Chakravarty D, Gao J, et al Prospective Comprehensive Molecular Characterization of Lung Adenocarcinomas for Efficient Patient Matching to Approved and Emerging Therapies. Cancer Discov. 2017;7(6):596–609. Epub 2017/03/25. 10.1158/2159-8290.CD-16-1337 28336552 PMC5482929

[12] Lee T, Lee B, Choi YL, Han J, Ahn MJ, Um SW. Non-small Cell Lung Cancer with Concomitant EGFR, KRAS, and ALK Mutation: Clinicopathologic Features of 12 Cases. J Pathol Transl Med. 2016;50(3):197–203. Epub 2016/04/19. 10.4132/jptm.2016.03.09 27086595 PMC4876086

[13] Sholl LM, Aisner DL, Varella-Garcia M, Berry LD, Dias-Santagata D, Wistuba II, et al Multi-institutional Oncogenic Driver Mutation Analysis in Lung Adenocarcinoma: The Lung Cancer Mutation Consortium Experience. Journal of thoracic oncology: official publication of the International Association for the Study of Lung Cancer. 2015;10(5):768–77. Epub 2015/03/05. 10.1097/JTO.0000000000000516 25738220 PMC4410843

[14] Won JK, Keam B, Koh J, Cho HJ, Jeon YK, Kim TM, et al Concomitant ALK translocation and EGFR mutation in lung cancer: a comparison of direct sequencing and sensitive assays and the impact on responsiveness to tyrosine kinase inhibitor. Annals of oncology: official journal of the European Society for Medical Oncology. 2015;26(2):348–54. Epub 2014/11/19. 10.1093/annonc/mdu530 . 25403583

[15] Yang JJ, Zhang XC, Su J, Xu CR, Zhou Q, Tian HX, et al Lung cancers with concomitant EGFR mutations and ALK rearrangements: diverse responses to EGFR-TKI and crizotinib in relation to diverse receptors phosphorylation. Clinical cancer research: an official journal of the American Association for Cancer Research. 2014;20(5):1383–92. Epub 2014/01/21. 10.1158/1078-0432.CCR-13-0699 . 24443522

[16] Zheng G, Tseng LH, Chen G, Haley L, Illei P, Gocke CD, et al Clinical detection and categorization of uncommon and concomitant mutations involving BRAF. BMC Cancer. 2015;15:779 Epub 2015/10/27. 10.1186/s12885-015-1811-y 26498038 PMC4619530

[17] Chong CR, Janne PA. The quest to overcome resistance to EGFR-targeted therapies in cancer. Nature medicine. 2013;19(11):1389–400. 10.1038/nm.3388 24202392 PMC4049336

[18] Lynch TJ, Bell DW, Sordella R, Gurubhagavatula S, Okimoto RA, Brannigan BW, et al Activating mutations in the epidermal growth factor receptor underlying responsiveness of non-small-cell lung cancer to gefitinib. The New England journal of medicine. 2004;350(21):2129–39. 10.1056/NEJMoa040938 . 15118073

[19] Adderley H, Blackhall FH, Lindsay CR. KRAS-mutant non-small cell lung cancer: Converging small molecules and immune checkpoint inhibition. EBioMedicine. 2019;41:711–6. Epub 2019/03/11. 10.1016/j.ebiom.2019.02.049 30852159 PMC6444074

[20] Ikediobi ON, Davies H, Bignell G, Edkins S, Stevens C, O’Meara S, et al Mutation analysis of 24 known cancer genes in the NCI-60 cell line set. Mol Cancer Ther. 2006;5(11):2606–12. Epub 2006/11/08. 10.1158/1535-7163.MCT-06-0433 17088437 PMC2705832

[21] Kondo Y, Hayashi K, Kawakami K, Miwa Y, Hayashi H, Yamamoto M. KRAS mutation analysis of single circulating tumor cells from patients with metastatic colorectal cancer. BMC Cancer. 2017;17(1):311 Epub 2017/05/05. 10.1186/s12885-017-3305-6 28468669 PMC5415811

[22] Raimbourg J, Joalland MP, Cabart M, de Plater L, Bouquet F, Savina A, et al Sensitization of EGFR Wild-Type Non-Small Cell Lung Cancer Cells to EGFR-Tyrosine Kinase Inhibitor Erlotinib. Mol Cancer Ther. 2017;16(8):1634–44. Epub 2017/05/20. 10.1158/1535-7163.MCT-17-0075 . 28522592

[23] Ran FA, Hsu PD, Lin CY, Gootenberg JS, Konermann S, Trevino AE, et al Double nicking by RNA-guided CRISPR Cas9 for enhanced genome editing specificity. Cell. 2013;154(6):1380–9. 10.1016/j.cell.2013.08.021 23992846 PMC3856256

[24] Ran FA, Hsu PD, Wright J, Agarwala V, Scott DA, Zhang F. Genome engineering using the CRISPR-Cas9 system. Nature protocols. 2013;8(11):2281–308. 10.1038/nprot.2013.143 24157548 PMC3969860

[25] Schneider CA, Rasband WS, Eliceiri KW. NIH Image to ImageJ: 25 years of image analysis. Nat Methods. 2012;9(7):671–5. Epub 2012/08/30. 10.1038/nmeth.2089 22930834 PMC5554542

[26] Chiang TW, le Sage C, Larrieu D, Demir M, Jackson SP. CRISPR-Cas9(D10A) nickase-based genotypic and phenotypic screening to enhance genome editing. Scientific reports. 2016;6:24356 10.1038/srep24356 27079678 PMC4832145

[27] Lauand C, Rezende-Teixeira P, Cortez BA, Niero EL, Machado-Santelli GM. Independent of ErbB1 gene copy number, EGF stimulates migration but is not associated with cell proliferation in non-small cell lung cancer. Cancer cell international. 2013;13(1):38 10.1186/1475-2867-13-38 23631593 PMC3655000

[28] Ettinger DS, Wood DE, Aisner DL, Akerley W, Bauman J, Chirieac LR, et al Non-Small Cell Lung Cancer, Version 5.2017, NCCN Clinical Practice Guidelines in Oncology. Journal of the National Comprehensive Cancer Network: JNCCN. 2017;15(4):504–35. 10.6004/jnccn.2017.0050 . 28404761

[29] Reck M, Popat S, Reinmuth N, De Ruysscher D, Kerr KM, Peters S, et al Metastatic non-small-cell lung cancer (NSCLC): ESMO Clinical Practice Guidelines for diagnosis, treatment and follow-up. Annals of oncology: official journal of the European Society for Medical Oncology. 2014;25 Suppl 3:iii27–39. 10.1093/annonc/mdu199 . 25115305

[30] Gandhi J, Zhang J, Xie Y, Soh J, Shigematsu H, Zhang W, et al Alterations in genes of the EGFR signaling pathway and their relationship to EGFR tyrosine kinase inhibitor sensitivity in lung cancer cell lines. PloS one. 2009;4(2):e4576 10.1371/journal.pone.0004576 19238210 PMC2642732

[31] Tracy S, Mukohara T, Hansen M, Meyerson M, Johnson BE, Janne PA. Gefitinib induces apoptosis in the EGFRL858R non-small-cell lung cancer cell line H3255. Cancer research. 2004;64(20):7241–4. 10.1158/0008-5472.CAN-04-1905 . 15492241

[32] Jakobsen JN, Santoni-Rugiu E, Grauslund M, Melchior L, Sorensen JB. Concomitant driver mutations in advanced EGFR-mutated non-small-cell lung cancer and their impact on erlotinib treatment. Oncotarget. 2018;9(40):26195–208. Epub 2018/06/15. 10.18632/oncotarget.25490 29899852 PMC5995236

[33] Zhang H, Bai H, Yang X, Zhong J, An T, Zhao J, et al Clinical outcome of epidermal growth factor receptor-tyrosine kinase inhibitors therapy for patients with overlapping kirsten rat sarcoma 2 viral oncogene homolog and epidermal growth factor receptor gene mutations. Thorac Cancer. 2016;7(1):24–31. Epub 2016/01/28. 10.1111/1759-7714.12266 26813477 PMC4718136

[34] Linardou H, Dahabreh IJ, Kanaloupiti D, Siannis F, Bafaloukos D, Kosmidis P, et al Assessment of somatic k-RAS mutations as a mechanism associated with resistance to EGFR-targeted agents: a systematic review and meta-analysis of studies in advanced non-small-cell lung cancer and metastatic colorectal cancer. Lancet Oncol. 2008;9(10):962–72. Epub 2008/09/23. 10.1016/S1470-2045(08)70206-7 . 18804418

[35] Mao C, Qiu LX, Liao RY, Du FB, Ding H, Yang WC, et al KRAS mutations and resistance to EGFR-TKIs treatment in patients with non-small cell lung cancer: a meta-analysis of 22 studies. Lung Cancer. 2010;69(3):272–8. Epub 2009/12/22. 10.1016/j.lungcan.2009.11.020 . 20022659

[36] Papadimitrakopoulou V, Lee JJ, Wistuba II, Tsao AS, Fossella FV, Kalhor N, et al The BATTLE-2 Study: A Biomarker-Integrated Targeted Therapy Study in Previously Treated Patients With Advanced Non-Small-Cell Lung Cancer. Journal of clinical oncology: official journal of the American Society of Clinical Oncology. 2016;34(30):3638–47. Epub 2016/08/03. 10.1200/JCO.2015.66.0084 27480147 PMC5065110

[37] Rulli E, Marabese M, Torri V, Farina G, Veronese S, Bettini A, et al Value of KRAS as prognostic or predictive marker in NSCLC: results from the TAILOR trial. Annals of oncology: official journal of the European Society for Medical Oncology. 2015;26(10):2079–84. Epub 2015/07/26. 10.1093/annonc/mdv318 . 26209642

[38] Engelman JA, Mukohara T, Zejnullahu K, Lifshits E, Borras AM, Gale CM, et al Allelic dilution obscures detection of a biologically significant resistance mutation in EGFR-amplified lung cancer. The Journal of clinical investigation. 2006;116(10):2695–706. Epub 2006/08/15. 10.1172/JCI28656 16906227 PMC1570180

[39] Yang JC, Wu YL, Schuler M, Sebastian M, Popat S, Yamamoto N, et al Afatinib versus cisplatin-based chemotherapy for EGFR mutation-positive lung adenocarcinoma (LUX-Lung 3 and LUX-Lung 6): analysis of overall survival data from two randomised, phase 3 trials. Lancet Oncol. 2015;16(2):141–51. Epub 2015/01/16. 10.1016/S1470-2045(14)71173-8 . 25589191

[40] Kruspig B, Monteverde T, Neidler S, Hock A, Kerr E, Nixon C, et al The ERBB network facilitates KRAS-driven lung tumorigenesis. Sci Transl Med. 2018;10(446). Epub 2018/06/22. 10.1126/scitranslmed.aao2565 29925636 PMC6881183

[41] Moll HP, Pranz K, Musteanu M, Grabner B, Hruschka N, Mohrherr J, et al Afatinib restrains K-RAS-driven lung tumorigenesis. Sci Transl Med. 2018;10(446). Epub 2018/06/22. 10.1126/scitranslmed.aao2301 . 29925635 PMC7610658

