# Supplementary figures and images for: Influence of EGFR-activating mutations on sensitivity to tyrosine kinase inhibitors in a KRAS mutant non-small cell lung cancer cell line
Source: PLoS One. 2020 Mar 4;15(3):e0229712. doi: 10.1371/journal.pone.0229712 (PMC7055889; doi:10.1371/journal.pone.0229712)

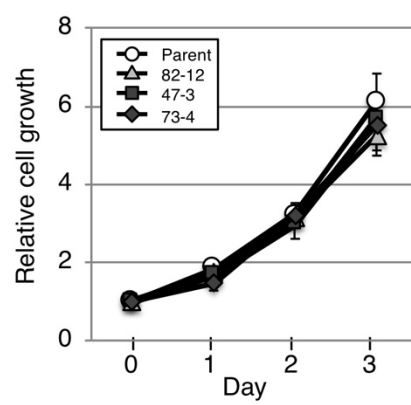

**Fig. S1**

Supplement: S1 Fig — Cells were seeded at 100000 cells/well in 6-well plate. The numbers of viable cells were stained by trypan blue and counted at day 0 (next day), 1, 2, and 3. (PDF) [file pone.0229712.s001.pdf]

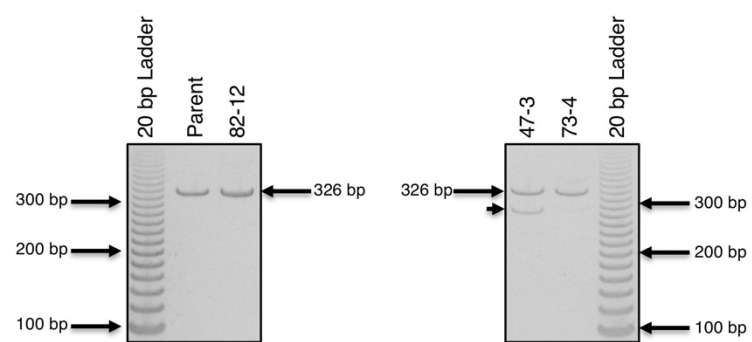

**Fig. S2**

Supplement: S2 Fig — Expressions of EGFR mRNAs containing intact and indel forms were analyzed by PCR using specific primer sets, which were designed around indel regions. Arrow: predicted PCR product, arrow head: 51 nt-deleted product. (PDF) [file pone.0229712.s002.pdf]

**A**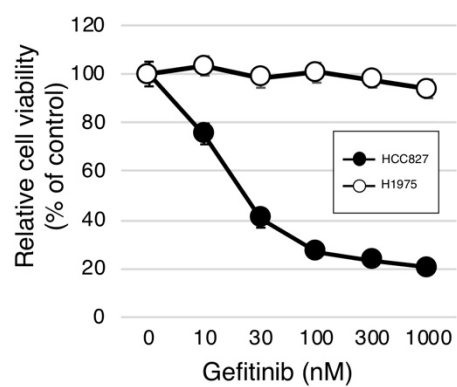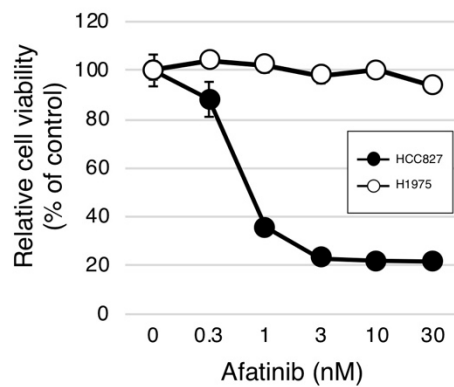**B**

| IC50           | HCC827 | H1975  |
|----------------|--------|--------|
| Gefitinib (nM) | 24.6   | > 1000 |
| Afatinib (nM)  | 0.81   | > 30   |

**C**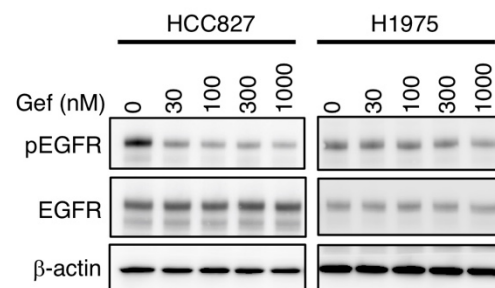**Fig. S3**

Supplement: S3 Fig — A. Cells were plated at 1 ´ 103 cells/well in 96-well plates and treated with gefitinib (10 to 1000 nM) and afatinib (0.3 to 30 nM) for 72 h. Cell viability was measured by the WST assay. B. The IC50 of gefitinib and afatinib is shown. C. HCC827 or H1975 cells were treated with the indicated concentration of gefitinib for 4 h. Protein expression was determined by immunoblotting using anti-phospho-EGFR (Y1068), anti-EGFR, and β- actin antibodies. (PDF) [file pone.0229712.s003.pdf]

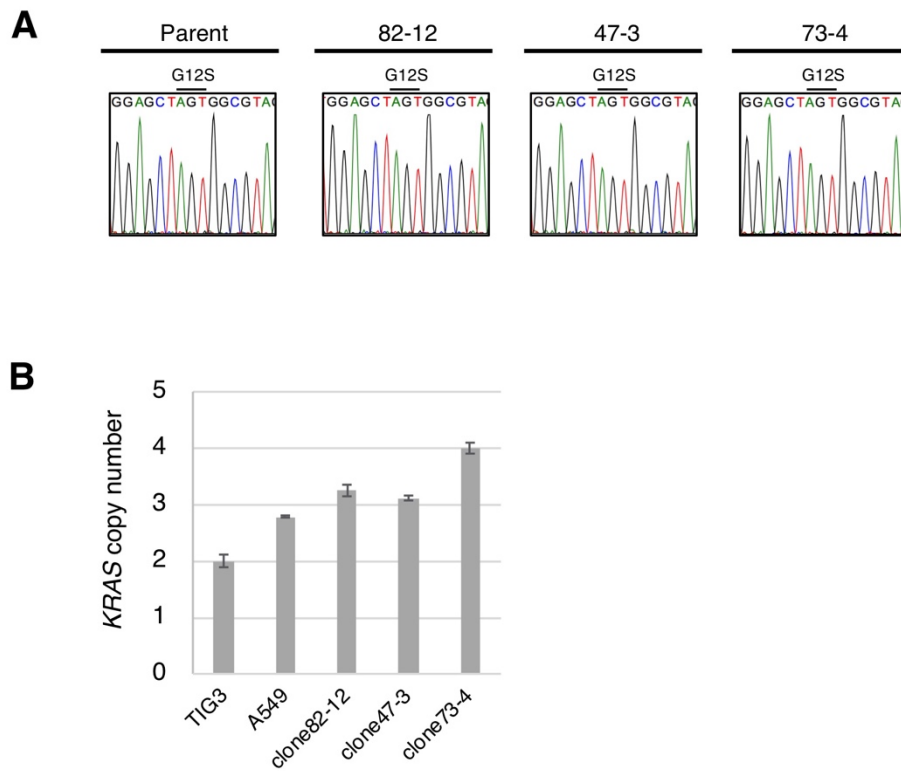

**Fig. S4**

Supplement: S4 Fig — A. Homozygous KRAS G12S mutation in each clone was validated by Sanger sequence. B. The KRAS copy number was determined by quantitative PCR using the diploid cell TIG3 as a control. (PDF) [file pone.0229712.s004.pdf]
