# Supplementary material for: Influence of EGFR-activating mutations on sensitivity to tyrosine kinase inhibitors in a KRAS mutant non-small cell lung cancer cell line
Source: PLoS One. 2020 Mar 4;15(3):e0229712. doi: 10.1371/journal.pone.0229712 (PMC7055889; doi:10.1371/journal.pone.0229712)
Supplement: S1 Methods — (PDF) [file pone.0229712.s005.pdf]

Supporting information

Methods

mRNA expression analysis

Total RNA was prepared from cells using the RNeasy mini kit (Qiagen). First-strand cDNA was synthesized from 1 μg total RNA with an oligo-dT primer using the SuperScript III First-Strand Synthesis System (Invitrogen). To detect the expression of indel form of *EGFR* mRNA in clone 47-3 and 73-4, the forward and reverse primer were designed at upstream or downstream of indel region, respectively: forward, 5'-GCTCCCAGTACCTGCTCAAC-3' and reverse, 5'- GATTCCGTCATATGGCTTGG-3'.

KRAS copy number analysis

Genomic DNA was extracted using the DNeasy Blood & Tissue Kit (Qiagen). Quantitative real-time PCR was performed with an ABI Prism 7300 sequence detection system (Applied Biosystems, Foster City, CA) using SYBR GreenER (Invitrogen) with KRAS-specific primers: forward, 5'-TTTGAGAGCCTTTAGCCGCC-3' and reverse, 5'-TCCAGTTGACTGCAGACGTG-3'. The relative copy number to the control in TIG-3 (diploid) was calculated using the comparative Ct method.
